# Supplementary material for: High-Resolution Computed Tomography (HRCT) Reflects Disease Progression in Patients with Idiopathic Pulmonary Fibrosis (IPF): Relationship with Lung Pathology
Source: J Clin Med. 2019 Mar 22;8(3):399. doi: 10.3390/jcm8030399 (PMC6463252; doi:10.3390/jcm8030399)
Supplement: Supplementary file 1 [file jcm-08-00399-s001.pdf]

Supplementary Material

High-Resolution Computed Tomography (HRCT) Reflects Disease Progression in Patients with Idiopathic Pulmonary Fibrosis (IPF): Relationship with Lung Pathology

Elisabetta Cocconcelli [1,†], Elisabetta Balestro [1,†], Davide Biondini [1], Giulio Barbiero [2], Roberta Polverosi [3], Fiorella Calabrese [1], Federica Pezzuto [1], Donato Lacedonia [4], Federico Rea [1], Marco Schiavon [1], Erica Bazzan [1], Maria Pia Foschino Barbaro [4], Graziella Turato [1], Paolo Spagnolo [1], Manuel G. Cosio [1,5,‡] and Marina Saetta [1,*,‡]

1 Department of Cardiac, Thoracic, Vascular Sciences and Public Health, University of Padova and Padova City Hospital, 35128 Padova, Italy; ecocconcelli@icloud.com (E.C.); Elisabetta.balestro@aopd.veneto.it (E.B.); dav.biondini@gmail.com (D.B.); fiorella.calabrese@unipd.it (F.C.); federica.pezzuto@libero.it (F.P.); federico.rea@unipd.it (F.R.); marco.schiavon@unipd.it (M.S.); erica.bazzan@unipd.it (E.B.); graziella.turato@unipd.it (G.T.); paolo.spagnolo@unipd.it (P.S.); marina.saetta@unipd.it (M.S.)

2 Institute of Radiology, Department of Medicine, University of Padova, 35128 Padova, Italy; giulio.barbiero@aopd.veneto.it

3 Istituto Diagnostico Antoniano - Affidea, 35100 Padova, Italy; rpolve@libero.it (R.P.)

4 Department of Medical and Surgical Sciences, University of Foggia, Policlinico "OO. Riuniti", 71122 Foggia, Italy; donatolacedonia@gmail.com (D.L.); mariapia.foschino@unifg.it (M.P.F.B.)

5 Meakins Christie Laboratories, Respiratory Division, McGill University, 65591 Montreal, QC, Canada; Manuel.cosio@mcgill.ca (M.G.C.)

* Correspondence: marina.saetta@unipd.it; Tel.: + 39 0498213704

† These two authors contributed equally to this work as first authors.

‡ These two authors contributed equally to this work as senior authors.

1. Methods

1.1. Study Population

This is a longitudinal study in which we analysed a well characterized cohort of IPF patient with a long clinical, functional and radiological follow up, referred in our transplant centre between 2011 and 2014 and before starting antifibrotic treatment.

For each patient the diagnosis of IPF was made in accordance with the last ATS/ERS/JRS/ALAT guidelines [1,2], either by clinical-radiological diagnosis (28 patients) or clinical-radiological-histological diagnosis (21 patients). Patients with a clear history of environmental or occupational exposure, and with clinical or serological data suggestive for a connective tissue disease were excluded.

For each patient the annual rate of decline in FVC% pred. was used to categorize the disease progression as slow (decline in FVC% pred. <10% per year) or rapid (decline in FVC% pred. ≥ 10% per year). Negative values of annual FVC% pred. and FVC ml decline during the follow-up indicated improvement.

A HRCT was available at diagnosis ($HRCT_1$) for all patients. Twenty-one patients (43%) had a second HRCT ($HRCT_2$), after a median of 17 (range 5-87) months of follow-up, and the clinical and functional data of this subgroup are shown in Table S1.

Table S1. Demographics and clinical characteristics of the 21 subjects with available follow up $HRCT_2$ (of which 12 slow and 9 rapid progressors)

	**All population (n=21)**	**Slow progressors (n=12)**	**Rapid progressors (n=9)**	***p*-value**
Male – n (%)	16 (76)	8 (67)	8 (89)	0.33
Age at diagnosis – years	58 (40-73)	58 (42-73)	58 (40-64)	0.88
Smoking history – pack years	13 (0-57)	13 (0-57)	17 (0-30)	0.96
• Current – n (%)	1 (5)	1 (8)	0 (0)	1
• Former – n (%)	17 (82)	9 (75)	8 (89)	0.6
• Non smokers – n (%)	3 (14)	2 (17)	1 (11)	1
Symptoms duration at diagnosis – months	24 (1-240)	23 (1-240)	24 (2-29)	0.43
Radiological diagnosis – n (%)	9 (43)	5 (42)	4 (44)	1
FVC at diagnosis – L	2.03 (1.75-4.06)	2.06 (1.84-4.06)	1.99 (1.75-3.49)	0.59
FVC at diagnosis – %pred.	69 (46-109)	73 (49-109)	59 (46-86)	0.24
DL_{CO} at diagnosis – %pred.	51 (13-106)	43 (37-106)	53 (13-97)	0.90
FVC decline per year – ml	210 (-330-1440)	95 (-330-380)	660 (331-1440)	0.0004
FVC decline per year – %pred.	9 (-30-29)	3 (-30-9)	16 (11-29)	0.0002
Time between $HRCT_1$ and $HRCT_2$ – months	17 (5-87)	24 (6-87)	11 (5-40)	0.03
Patients undergoing transplant – n (%)	6 (29)	4 (33)	2 (22)	0.55
Patients who died – n (%)	13 (62)	6 (50)	7 (78)	0.19

Values are expressed as numbers and (%) or medians and ranges. *p*-values refers to comparison between slow and rapid progressors.

At diagnosis, sex, age, smoking history and respiratory function (FVC both % predicted and millilitres-ml) were similar in slow and rapid progressors. The radiological follow-up period was longer in *slow* than in rapid progressors (median; range: 24; 6-87 months *vs* 11; 5-40 months; p=0.03).

$HRCT_1$ and $HRCT_2$ were scored blindly and independently by two expert thoracic radiologists by using a quantitative scale, as previously described [8]. This score is made up by the assessment of ground glass opacities (alveolar score, AS%) and fibrotic extent (interstitial score, IS%) for each lung lobe, analyzing each series with axial slice thickness ≤ 2.5 mm and a limited slice spacing ≤ 10 mm. After each individual lobe was scored, the final result of AS% and IS% for the whole lung was expressed as mean value of the five lobes (AS and IS, respectively).

In the twenty-one IPF patient in whom a second HRCT and lung function assessment were available, we studied the correlation between the radiological changes and FVC decline by calculating the change per month of Alveolar Score (ΔAS/month) and Interstitial Score (ΔIS/month), and the change per month in FVC (ΔFVC% pred./month and ΔFVC ml/month) in the period from $HRCT_1$ to $HRCT_2$.

We express the radiological changes per month to normalise the differences in timing between $HRCT_1$ and $HRCT_2$ in the slow and rapid progressors.

1.2 Pathological analysis

Thirteen of the forty-nine patients underwent lung transplantation during the follow up. Clinical and functional data of this subgroup are shown in Table S2.

Table S2. Demographics and clinical features of the 13 subjects undergoing lung transplantation (of which 6 slow and 7 rapid progressors)

	**Entire population (n=13)**	**Slow progressors (n=6)**	**Rapid progressors (n=7)**	***p*-value**
Male – n (%)	13 (100)	6 (100)	7 (100)	1
Age at diagnosis – years	54 (33-64)	59 (56-80)	52 (33-64)	0.56
Smoking history – pack years	22 (0-92)	26 (0-57)	19 (0-92)	0.59
• Current – n (%)	0 (0)	0 (0)	0 (0)	1
• Former – n (%)	11 (85)	5 (84)	6 (86)	1
• Non smokers – n (%)	2 (15)	1 (16)	1 (14)	1
Symptoms duration at diagnosis – months	10 (1-48)	12 (1-48)	10 (1-44)	0.83
Radiological diagnosis – n (%)	7 (54)	5 (83)	2 (29)	0.1
FVC at diagnosis – L	2 (1.28-3.17)	2.17 (1.28-3.17)	2.09 (1.75-2.51)	0.9
FVC at diagnosis – %pred.	59 (36-86)	50 (36-74)	62 (52-86)	0.045
DL_{CO} at diagnosis – %pred.	40 (10-97)	40 (28-97)	36 (10-54)	0.2
FVC decline per year – ml	444 (0-1498)	140 (0-320)	783 (588-1498)	0.0039
FVC decline per year – %pred.	11 (1-27)	4 (1-9)	16.3 (11-27)	0.0082
Time between HRCT and transplantation – months	1 (0-20)	3 (0-14)	4 (0-20)	0.9
Patients who died – n (%)	10 (77)	5 (83)	5 (71)	1

Values are expressed as numbers and (%) or median and ranges. *p*-values refers to comparison between slow and rapid progressors.

At diagnosis, sex, age, smoking history and FVC values (both % predicted and millilitres-ml) were similar in slow and rapid progressors. Time between the last HRCT performed and lung transplantation were similar in slow and rapid progressors.

The native lungs were fixed in formalin by airway perfusion and samples were obtained and embedded in paraffin. Sections 5 μm thick were cut and stained for histological and immunohistochemical analysis, as previously described [3].

Fibroblastic foci were counted in sections stained with hematoxylin–eosin and expressed as number of fibroblastic foci/mm^2 of area examined. Cellular infiltrate including total leukocytes ($CD45^+$), neutrophils, macrophages ($CD68^+$), and total lymphocytes calculated as sum of $CD4^+$, $CD8^+$ T lymphocytes and $CD20^+$ B lymphocytes was identified by immunohistochemistry as previously described [17,18]. Each inflammatory cell type was quantified in 20 non-overlapping high-power fields per slide and expressed as cells/mm^2 of area examined.

1.3 Statistical analysis

Categorical variables were described as absolute (n) and relative values (%) and continuous variables were described as median and range. To compare demographic and pathological data between rapid and slow progressors Chi square test and Fisher's exact test ($n < 5$) for categorical variables and Mann-Whitney U test for continuous variables were used. To evaluate the difference between $HRCT_1$ and $HRCT_2$, we performed a Wilcoxon (paired test) analysis.

The relationship between ΔAS/month, ΔIS/month and ΔFVC ml/month and the relationship between AS and IS scores with inflammatory infiltrates and FF were evaluated using Spearman's rank correlation. *Adjusted p*-values for multiple comparisons were calculated using the Holm method. The inter-observed agreement between the two radiologists in the scoring of the abnormality was evaluated by kappa statistic measure. All data were analyzed using SPSS Software version 25.0 (New York, NY, US: IBM Corp. USA). *p*-values < 0.05 were considered statistically significant.

2. Results

Radiologic analyses for different *regions* (upper and lower lobes) and for total lung are shown in Table S3. In $HRCT_1$, Alveolar Score, considered both separated for lung regions or all together in total lung, was significantly greater in rapid than slow progressors. In $HRCT_1$, Interstitial Score, considered both separated for lung regions or all together in total lung, was similar between rapid and slow progressors.

Table S3. Alveolar Score (AS) and Interstitial Score (IS) of $HRCT_1$ in the entire population (n=49), of which 30 slow and 19 rapid progressors.

	**Entire population (n=49)**	**Slow progressors (n=30)**	**Rapid progressors (n=19)**	***p*-value**
$HRCT_1$ Total lung AS - %	10 (0-84)	3 (0-75)	21 (0-4)	0.008
• Upper region AS - %	5 (0-82)	2 (0-70)	17 (0-82)	0.02
• Lower region AS - %	10 (0-100)	5 (0-100)	23 (0-88)	0.006
$HRCT_1$ Total lung IS - %	28 (1-84)	27 (1-84)	30 (9-75)	0.85
• Upper region IS - %	18 (0-82)	19 (0-82)	17 (0-70)	0.63
• Lower region IS - %	38 (1-100)	38 (1-100)	38 (5-88)	0.74

Values are expressed as medians and range. *p*-values refers to comparison between slow and rapid progressors.

Table S4. Alveolar Score (AS) and Interstitial Score (IS) of both h HRCT scans ($HRCT_1$ and $HRCT_2$) in the entire population (n=49), of which 30 slow and 19 rapid progressors.

	Entire population (n=21)		*p*-value	Slow progressors (n=12)		*p*-value	Rapid progressors (n=9)		*p*-value
	$HRCT_1$	$HRCT_2$		$HRCT_1$	$HRCT_2$		$HRCT_1$	$HRCT_2$	
AS	7 (0-84)	13 (0-82)	0.02	6 (0-75)	12 (0-63)	0.21	32 (0-84)	47 (0-82)	0.03
IS	26 (6-75)	40 (9-84)	0.0009	25 (6-62)	29 (9-84)	0.03	26 (9-75)	56 (12-80)	0.01

Values are expressed as medians and ranges. *p*-values refers to comparison between $HRCT_1$ and $HRCT_2$.

2.1 Functional-radiological correlations

The positive correlation between ΔFVC and ΔIS was confirmed when the change in FVC was expressed as ΔFVC% predicted (r=0.55, p=0.01). When stratified in slow and rapid progressors, the correlation was equally confirmed in the rapid group (r=0.87, p=0.01), but not in the slow group (r=0.27, p=0.38). Again, the correlation between ΔFVC% pred./month and ΔAS/month was not significant (r= 0.11; p=0.64).
